# Mechanisms of root exudates and soil microbial responses to nitrogen reduction and companion cropping for tomato yield increase and quality improvement

**DOI:** 10.3389/fpls.2025.1642078

**Published:** 2025-11-18

**Authors:** Deyang Liu, Xingjia He, Yingxue Liu, Chaofan Sun, Chunjie Su, Yishan Lang, Xia Wu

**Affiliations:** College of Horticulture and Landscape, Heilongjiang Bayi Agricultural University, Daqing, Heilongjiang, China

**Keywords:** nitrogen reduction, tomato quality, companion cropping, bacterial diversity, root exudates

## Abstract

The utilization of nitrogen fertilizer in excess over an extended period in facility cultivation has been demonstrated to engender a decline in tomato yield and quality, thus becoming a bottleneck issue that restricts production. In order to explore the biological mechanisms of nitrogen reduction and companion planting patterns on tomato yield and quality, this study conducted a two-year pot experiment under different nitrogen application levels and planting patterns, multisystem analysis of tomato rhizosphere soil microbial communities and root secretions was performed using 16S Illumina MiSeq sequencing and LC-MS/MS mass spectrometry. Over two years, the yield of tomatoes grown using reduced nitrogen and companion planting increased by 34.26% and 35.54% compared to monoculture, and by 1.23% and 3.57% compared to the application of conventional nitrogen and companion planting. Nitrogen use efficiency increased by 9.81% and 11.36%, respectively. The nitrogen reduction and companion planting model increased the content of soluble sugars and lycopene, improved soil dehydrogenase and protease activity, and changed the composition of rhizosphere bacterial communities and root exudates. At all nitrogen application rates, companion planting increased the abundance of *Pseudomonas*. In the 30% nitrogen reduction and conventional nitrogen application systems, the abundance of differential metabolites such as methionine and pipecolic acid was significantly higher in companion crops than in tomato monoculture. On the other side, soil bacteria and root exudates form a complex network of interactions, in which rhizosphere bacteria such as *MND1* are positively correlated with *Sphingomonas*. In summary, the cultivation model of nitrogen reduction and companion planting of potato onions changed the composition of the tomato soil bacterial community and the metabolic pathways of root exudates, enhanced the activity of nitrogen conversion-related enzymes, and promoted the absorption and utilization of nitrogen nutrients by tomatoes, which provides a theoretical basis for increasing the yield and quality of tomatoes cultivated with 30% nitrogen reduction and companion planting of onions.

## 1 Introduction

Facility cultivation is of critical importance and value in meeting the growing global demand for vegetables. Tomatoes (*Solanum lycopersicum* L.) are among the most widely cultivated facility vegetables, with extensive cultivation occurring in both northern and southern China (Zhang P. et al., 2024). Tomatoes are rich in various nutrients, such as soluble protein, lycopene, and vitamin C, which give them strong antioxidant properties. They can reduce the risk of cancer and cardiovascular disease and have medicinal value (Gorni et al., 2022). In order to ensure the consistent production of high yields of tomatoes, it is imperative to recognize the stringent requirements of greenhouse-grown tomatoes for water and nutrients (Wu et al., 2024). The nitrogen application rate in China’s primary vegetable-producing regions is 1,000 kg ha^-1^ yr^-1^, which significantly exceeds the actual demand for vegetables. Excessive use of nitrogen fertilizers has been demonstrated to result in a number of detrimental outcomes, including soil degradation and nitrate accumulation. Furthermore, this can disrupt the balance of nutrients in the soil, leading to crop nutrient imbalances, reduced quality and lower yields (Li et al., 2017; Tang J. et al., 2023). Extensive research has shown that reducing nitrogen fertilizer application rates does not affect crop yields while maximizing nitrogen use efficiency (NUE). A 30% reduction in nitrogen is key to effective nitrogen fertilizer management (Keränen et al., 2015; Luo et al., 2018; Bai et al., 2025). On the other hand, long-term continuous cropping has become a major problem restricting tomato production, leading to deterioration of soil physical and chemical properties and imbalance in the microbial community structure, which in turn reduces crop productivity (Song et al., 2022). Many studies have shown that intercropping and companion planting can promote growth and increase productivity by utilizing complementary interactions between different species (Feng et al., 2021; Yang et al., 2022). Despite the focus of preceding studies on enhancing crop rotation, a systematic evaluation of the synergistic impacts of reduced nitrogen fertilization and companion planting has not been conducted. Therefore, this study investigated the close relationship between nitrogen reduction fertilization and tomato rhizosphere exudates, soil microorganisms, yield and quality under companion cropping conditions. This is of great significance for sustainable agricultural development through nitrogen reduction and efficiency improvement.

Soil microorganisms play an important role in maintaining agricultural ecosystems and sustainable production (Zhou and Wu, 2018). Soil microorganisms are closely related to nutrient cycling and largely determine soil properties and nitrogen supply capacity. At the same time, changes in soil physical and chemical properties cause changes in the soil microenvironment, which in turn affect the community composition and function of soil microorganisms (Delgado-Baquerizo et al., 2018). The symbiotic relationship between tomatoes and potato onions has been proven to promote plant growth and enhance resistance to diseases such as yellow wilt by changing the composition of rhizosphere microorganisms (Li N. et al., 2020). Although many studies have demonstrated that intercropping increases productivity through rhizosphere effects, there has been little research into the specific underground biochemical processes that generate these benefits. Reducing nitrogen application significantly increased soil bacterial community diversity and evenness, possibly due to the weakened dominance of nitrogen-sensitive groups, which in turn released ecological niches for less competitive species (Li et al., 2023). Therefore, the relationship between nitrogen fertilizer reduction, nitrogen nutrient utilization, and microbial community structure in companion cropping systems warrants further investigation.

The rhizosphere is a critical region in which nutrient cycling, the physical and chemical properties of the soil, and biological and environmental factors interact. Some root exudates can act as biological nitrification inhibitors, reducing soil nitrogen loss and improving plant nitrogen use efficiency (Sun et al., 2016). Root exudates are also an important medium through which plants interact with microbes (Berendsen et al., 2012). The formation of rhizosphere microbial communities is influenced by a number of factors, including plant diversity and community selection. However, it is also regulated by the chemical microenvironment, which is primarily composed of metabolites (Cheng et al., 2022). Stringlis et al. found that Arabidopsis roots secrete coumarin-derived myb72-regulated scopoletin into the rhizosphere, which shapes root-associated microbial communities by exerting selective antibacterial activity (Stringlis et al., 2018). Previous studies have primarily concentrated on the effects of nitrogen application rates or intercropping as single factors on crop rhizosphere microbial diversity. However, there is a paucity of research on the relationship between different nitrogen application rates and soil microbial and root metabolic levels in intercropping systems. Furthermore, the mechanisms by which root exudates interact with soil microorganisms and promote growth, increase yield, and improve quality of crop under different nitrogen application levels and companion plant systems remain unclear. Therefore, the present study focuses on in-depth research on root secretion-mediated nitrogen reduction, accompanied by increased yield and quality.

In order to investigate how companion planting of onion promotes tomato growth and improves fruit quality by shaping the rhizosphere microbial community and rhizosphere metabolites, we conducted a two-year pot experiment. First, we analyzed the effects of companion planting and nitrogen application on tomato growth, yield and quality, and investigated changes in soil enzyme activities related to nitrogen transformation in the rhizosphere. Concurrently, a comparison was made of the changes in soil microbial community structure and composition, as well as the composition and abundance of root-secreted metabolites under different treatments. Furthermore, the interactions between soil microbes and metabolites were explored through co-occurrence network analysis. We aim to clarify: (1) Evaluate whether nitrogen reduction and companion planting can improve tomato yield, quality, and nitrogen fertilizer utilization efficiency. (2) Clarify how nitrogen reduction and companion planting affect the composition and abundance of tomato root exudates, thereby influencing the structure of the rhizosphere microbial community and improving nitrogen absorption and utilization, and elucidate the biological mechanism by which nitrogen reduction and companion planting improve quality and yield.

## 2 Materials and methods

### 2.1 Description of test materials and sites

In this study, the tomato (*Solanum lycopersicum* L.) variety “Dongnong 708” and the potato onion variety (*Allium cepa* L. var.*aggregatum* G. Don) “Wuchang” were used as the main materials. The study site was located at the experimental base of Heilongjiang Bayi Agricultural Reclamation University (45°46′N, 124°19′E). The test soil is soil that has been used for continuous tomato cultivation for a period of four years. The fundamental physical and chemical properties of the soil are as follows: pH 8.3 (1:5, soil: water); soil EC 1.07 mS·cm^−1^; NH4^+^-N 7.61 mg·kg^−1^, NO3^−^-N 30.82 mg·kg^−1^ (Liu et al., 2022); AP 84.99 mg·kg^-1^, AK 152.3 mg·kg^-1^ (Duan et al., 2024); Organic matter (OM) 23.60 g· kg^−1^ (Zhan et al., 2024).

### 2.2 Experimental design and sampling

#### 2.2.1 Experimental design

**Figure 1:**
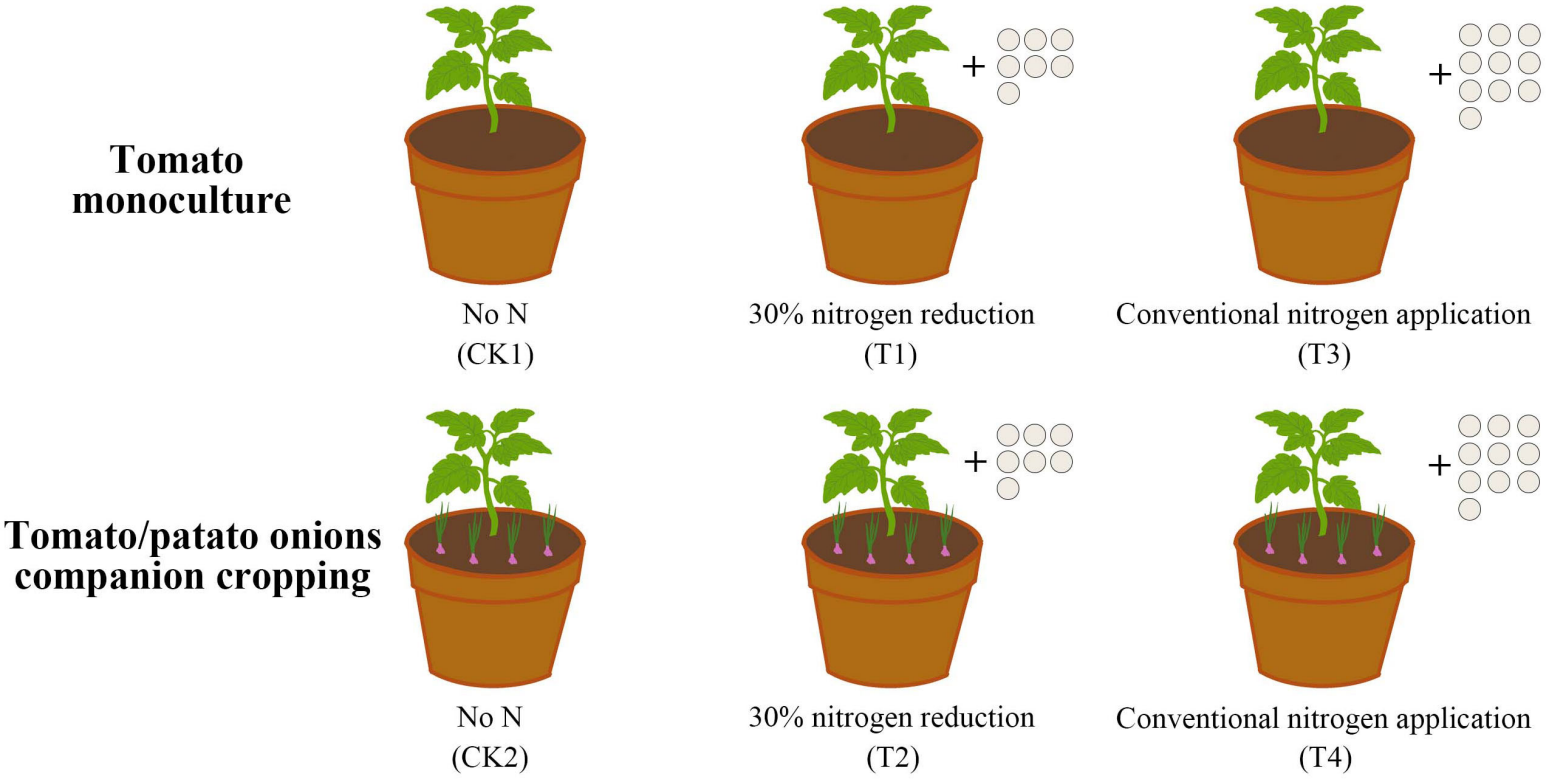
Cultivation pattern of tomato accompanied by potato onions and pattern of nitrogen application treatments.

A two-year pot culture experiment was conducted from April to August in 2023 and 2024. The conventional nitrogen application rate for tomatoes in local facilities (0.67 g/kg-1) and preliminary test results by the research team were taken into consideration in the design of this study, six treatments were established (**Table 1**), with three replicates per treatment and 10 pots per replicate, for a total of 180 pots, which were arranged randomly. Depending on the treatment, mix the fertilizer evenly with 7.5 kg of soil and place in pots (25 cm in diameter and 20 cm high). For single cropping, plant one tomato plant per pot. For intercropping, plant one tomato plant and four onion bulbs per pot, spacing the onion bulbs 5 cm apart and surrounding the tomato plants halfway around (**Figure 1**). Tomato seedings were transplanted on 22 May, with five leaves and one heart, and one leaf was left on the second fruit cluster and the tip was removed. According to the experimental design, urea at different concentrations and equal amounts of Ca(H_2_PO_4_)_2_·CaSO_4_ and K_2_SO_4_ were applied in two separate applications during the fruit enlargement stage and the green ripening stage. The growth indicators and quality of potted plants in 2024 were comparable to those in 2023. At 40 days, tomato rhizosphere soil samples were collected simultaneously to measure soil enzymes and microbial diversity, while metabolomic analysis was conducted on extracts from tomato plant root exudates.

**Table 1:** Experimental design.

Treatment	Cultivation system	Urea(g·kg^-1^)	Ca(H_2_PO_4_)_2_·CaSO_4_(g·kg^-1^)	K_2_SO_4_(g·kg^-1^)
CK1	Tomato monoculture	0	0.55	0.67
CK2	Tomato/potato onions companion planting	0	0.55	0.67
T1	Tomato monoculture	0.47	0.55	0.67
T2	Tomato/potato onions companion planting	0.47	0.55	0.67
T3	Tomato monoculture	0.67	0.55	0.67
T4	Tomato/potato onions companion planting	0.67	0.55	0.67

#### 2.2.2 Soil and plant sampling

At 40 days after tomato transplanting (initial fruiting stage), five plants were randomly selected from each replicate for measurement of growth indicators. The plant height was measured using a long straight ruler. The SPAD value and nitrogen content of the leaves were measured using a portable chlorophyll meter (HED-YD, Huoerde Electronic, China). The root-shaking method was employed to gently dislodge large clumps of soil wrapped around the roots, and a brush is then utilized to collect the soil adhering to the rhizosphere (Wang et al., 2009). To eliminate individual variation, three biological replicates (n = 3) were established for each treatment. For each replicate, root rhizosphere soil from five tomato pots was pooled to form a single sample, resulting in a total of 15 pots per treatment. Following filtration through a 2 mm sieve, one portion was stored at 4 °C for the purpose of measuring soil enzyme activity, and the other portion was stored at -80 °C for high-throughput sequencing of bacterial communities (Zhou and Wu, 2012). Another portion of tomato seedlings was rinsed with water to clean the roots, then washed three times with deionized water. The seedlings were placed in glass bottles for cultivation. Deionized water was added to the bottles until the roots were submerged, and the bottles were placed in an incubator for 6 hours. The water level was monitored and maintained. After the cultivation period, root exudates were collected. The final concentration of root exudates was adjusted to 1 g root fresh weight per 10 ml (1 g FW·10 ml^-1^) in water, filtered through two layers of 0.45 μm filter membranes, stored at -80 °C for analysis of root exudate metabolomics (Zhou et al., 2023). During red ripening stage, the yield per tomato plant was measured. For each treatment, five fruits from the second cluster were randomly selected, pulped and homogenized to constitute a single biological replicate. These samples were rapidly frozen in liquid nitrogen and stored at -40 °C for subsequent quality analysis. After harvesting the fruits, we collected the plant material, washed it, dried it with filter paper to remove moisture, blanched it at 105 °C for 30 minutes, dried it at 80 °C until constant weight, ground it, took 0.5 g of the dried plant material, nitrated it with H_2_SO_4_ and H_2_O_2_, and determined the total nitrogen content of the plant material (Wang et al., 2022). Nitrogen utilization efficiency (NUE, %) = Y/TN. Where, Y is the tomato yield (kg) and TN is the total N uptake of tomato (g/plant), TN = total nitrogen content (g/kg) × dry matter weight (kg/plant) (Chen et al., 2017).

### 2.3 Experimental methods

#### 2.3.1 Measurement of rhizosphere soil enzymes and tomato quality

Urease activity was determined using the phenol-sodium hypochlorite colorimetric method (Wang et al., 2019). The TTC colorimetric method was used to determine soil dehydrogenase activity (Wieczorek et al., 2015). The soil protease activity was determined using the indophenol colorimetric method. Fresh soil samples were mixed with casein solution, incubated at 37 °C for 24 hours, and then measured by colorimetry (Hou et al., 2021). Soil nitrate reductase activity was determined using the Guan Songyin-phenol disulfonic acid colorimetric method (Yang Z. et al., 2020). The soluble sugar content was determined using the anthrone colorimetric method, and the titratable acid was determined using the NaOH-phenolphthalein titration method (Bian et al., 2018). The content of soluble protein was determined using the Coomassie Brilliant Blue colorimetric method (Georgiou et al., 2008). The lycopene content was determined by the hexane-acetone-ethanol extraction method (Talpur et al., 2023). The vitamin C (VC) content of tomato fruits was determined using the titration method (Mao et al., 2024).

#### 2.3.2 Soil DNA extraction and Illumina MiSeq sequencing and data processing

Total DNA was extracted from soil sample using a soil DNA (MP Biomedicals, USA) kit, and the concentration, quality, and purity of the extracted DNA were determined using an ultramicro spectrophotometer (Thermo Fisher Scientific, NanoDrop2000) and 1% (w/v) agarose gel electrophoresis. Amplification sequencing was performed on the bacterial 16S rRNA gene using the V3-V4 regions of primers F (5 ‘-ACTCCTACGGGAGGCAGCA) -3’ and R (GGACTACHVGGGTWTCTAAT) (Feng et al., 2023). Illumina platform was used for paired-end sequencing of community DNA fragments. For the ASVs that were observable and their relative abundance, sequence length distribution statistics were utilized in R language scripts to statistically analyze the length distribution of high-quality sequences contained in all samples. For the 16S rRNA gene of bacteria, the classify-sklearn algorithm in QIIME2 (2019.04) was utilized to classify and annotate the feature sequences of each ASV based on the raw data from the Greengenes database (http://greengenes.lbl.gov) (Caporaso et al., 2010). Bacteria were classified using the GreenGenes (Version 135) database, and the microbial abundance at different phylogenetic levels (phylum, class, order, family, genus, and species) in each sample was assessed using Bracken (Bayesian Reestimation of Abundance after Classification with KrakEN) (Lu et al., 2017).

#### 2.3.3 Metabolomic analysis of root exudates

In order to analyze the metabolite profile of root exudates, 0.25 g of freeze-dried root exudate powder was added to 500 μL of pre-chilled extraction solution (methanol: acetonitrile:water = 2:2:1 (v/v)), and the mixture was then homogenized with a bead mill. The mixture was then extracted, incubated at -40 °C for 1 hour, and the sample was subjected to a centrifugation process at 12,000 rpm for 15 minutes. The superior portion of the specimen was collected. Use Vanquish (Thermo Fisher Scientific) to perform liquid chromatography separation of the target compounds using a Phenomenex Kinetex C18 column (2.1 mm × 50 mm, 2.6 μm). The Piseno Bio Sequencing Company performed optimization of UPLC-Q-TOF-MS/MS conditions and substance analysis. A comparison of the database was conducted to identify potential substances. The root exudate component samples were then screened using the target screening function of Masterview 1.0 software. The raw data was converted to mzXML format using ProteoWizard, and then peak alignment, retention time correction, and peak area extraction were performed using XCMS software. The data obtained from XCMS is first subjected to metabolite structure identification and data analysis. Principal component analysis (PCA) of the samples was performed using R 4.1.0 software. The differential metabolites were then subjected to screening based on two criteria: significance (*p* < 0.05) and differences in fold change (FC, FC ≥ 1.5 or ≤ 0.67) in univariate analysis. Using MetaboAnalyst 5.0 (https://www.metaboanalyst.ca/), Kyoto Gene and Genome Encyclopedia (KEGG) enrichment analysis was performed on differentially expressed metabolites based on Fisher’s exact test (Kanehisa et al., 2023).

### 2.4 Statistical analysis

Data were statistically analyzed using SPSS 23.0 relying on Turkey’s HSD, (*p* < 0.05). Bar graphs were made using Origin 2024 software. The α-diversity index of the bacterial community was calculated using QIIME2 (2019.4) software, plotted as a box-and-line plot using R 4.1.0 software with the ggplot2 package, including Chao1 index, Shannon index, and Simpson index. We employed non-metric multidimensional scaling (NMDS) and bray-curtis dissimilarity matrices to visualize bacterial and community structures. The results of NMDS analysis were examined using anosim and adonis based on the bray-curtis distance and 999 permutations. Using R 4.1.0 and the Python LefSe and ggtree packages, we conducted differential analysis of bacterial composition at all taxonomic levels under different treatments to identify robust differentially abundant species, i.e., biomarkers, between groups. Using R 4.1.0 and the Python LefSe and ggtree packages, we conducted differential analysis of bacterial composition at all taxonomic levels under different treatments to identify robust differentially abundant species, i.e., biomarkers, between groups. Principal component analysis (PCA) was utilized to compare the degree of similarity in root exudates diversity among different samples. The OPLS-DA model was used to screen for differential metabolite molecules between the two groups of samples, with VIP > 1 and *P* value < 0.05 as significant differential metabolite screening criteria. Based on the KEGG enrichment results, the degree of enrichment was measured by Rich factor, FDR value and the number of metabolites enriched to this metabolic pathway to compare the different metabolites of different metabolic pathways between the two groups of samples. Co-occurrence networks of soil differential metabolites with bacterial genus level were realized by Cytoscape 3.7.1 software (https://www.cytoscape.org/). Correlations between soil enzyme activities, microbial community composition, metabolite composition and tomato growth, yield and quality indicators were assessed by Mantel’s test (999 permutations) using R 4.1.0 software based on Euclidean distance matrix.

## 3 Results

### 3.1 Effect of nitrogen reduction and potato onions companion cropping on tomato growth and yield

The results of a two-year trial showed that the plant height, leaf nitrogen content, and dry weight of T2 were significantly higher than those of CK1 (**Table 2**, *p* < 0.05). The two-year tomato yields of T2 were significantly higher than that of CK1 by 34.26% and 35.54%. At a 30% nitrogen reduction level, the SPAD value and plant dry weight of T2 were higher than those of T1, and reaching a significant level in 2023, with a significant increase in yield of 35.51% (*p* < 0.05). The yield of T2 in 2024 was found to be significantly higher than that of T1 by 15.49% (*p* < 0.05), and higher than that of T4 in the two years by 1.23% and 3.57%, respectively. In the two years trial, T2 had the highest NUE, higher than the other treatment, and increased by 9.81% and 11.36% compared to T4.

**Table 2:** Effects of nitrogen reduction and potato onion associated with tomato plant growth.

Year	Treatment	Plant height (cm)	Stemdia meters (mm)	SPAD	Leaf nitrogen (mg·g^-1^)	Plant total nitrogen (mg·g^-1^)	Plant dry weight (g)	Tomato yield (kg·plant^-1^)	NUE(%)
2023	CK1	53.5 ± 1.85d	10.62 ± 0.35c	47.083 ± 2.75d	16.33 ± 0.76b	47.28 ± 3.13b	24.84 ± 2.68c	1.08 ± 0.09b	/
	CK2	57.2 ± 1.34cd	12.15 ± 0.5bc	54.35 ± 3.09cd	18.73 ± 0.94ab	51.57 ± 3.78ab	28.71 ± 0.86abc	1.14 ± 0.06b	/
	T1	60.5 ± 1.74abc	12.72 ± 0.53ab	57.03 ± 2.25bc	18.9 ± 1.33ab	49.77 ± 1.37ab	27.05 ± 1.8bc	1.07 ± 0.09b	17.3 ± 0.76b
	T2	62.25 ± 1.11ab	13.71 ± 0.4a	67 ± 2.92a	21.5 ± 1.06a	56.62 ± 4.94ab	33.76 ± 1.25a	1.45 ± 0.07a	23.25 ± 1.9a
	T3	59 ± 1.68bcd	12.65 ± 0.47ab	64.68 ± 2.9ab	18.43 ± 1.27ab	56.95 ± 0.19ab	32.5 ± 0.4ab	1.26 ± 0.05ab	19.64 ± 0.66ab
	T4	64.5 ± 1.55a	12.2 ± 0.44abc	63.53± 4.12abc	21.55 ± 1.72a	61.25 ± 4.65a	32.89 ± 0.33ab	1.4 ± 0.09a	21.17 ± 1.76ab
2024	CK1	59.1 ± 1.22b	10.35 ± 0.47b	43 ± 2.93b	18.20 ± 0.33b	47.27 ± 2.49c	15.73 ± 1.26c	1.21 ± 0.02b	/
	CK2	61.5 ± 1.43b	11.21 ± 0.38ab	44.1 ± 4.1b	20.2 ± 0.18ab	48.34 ± 1.68bc	21.974 ± 1.73b	1.29 ± 0.02b	/
	T1	64.6 ± 2.64ab	11.51 ± 0.59ab	48.16 ± 3.82b	19.28 ± 0.51ab	51.71 ± 0.8abc	21.068 ± 0.79b	1.42 ± 0.1b	18.8 ± 1.01b
	T2	67.8 ± 1.69a	10.88 ± 0.1ab	56.18 ± 3.75ab	23.10 ± 0.97a	54.69 ± 1.94a	25.58 ± 2.71ab	1.64 ± 0.06a	24.31 ± 1.26a
	T3	60.4 ± 1.63b	11.75 ± 0.62ab	52.74 ± 1.88ab	17.22 ± 0.38b	56.27 ± 2.05a	27.702 ± 1.53a	1.66 ± 0.08a	19.79 ± 0.64b
	T4	63 ± 1.63b	12.03 ± 0.5a	62.96 ± 1.98a	20.08 ± 1.32ab	54.32 ± 2.02ab	25.64 ± 0.92ab	1.62 ± 0.04a	21.83 ± 0.69ab

Different letters indicate the statistically significant differences among treatments (P<0.05).

### 3.2 Effect of nitrogen reduction and potato onions companion cropping on tomato quality

**Figure 2:**
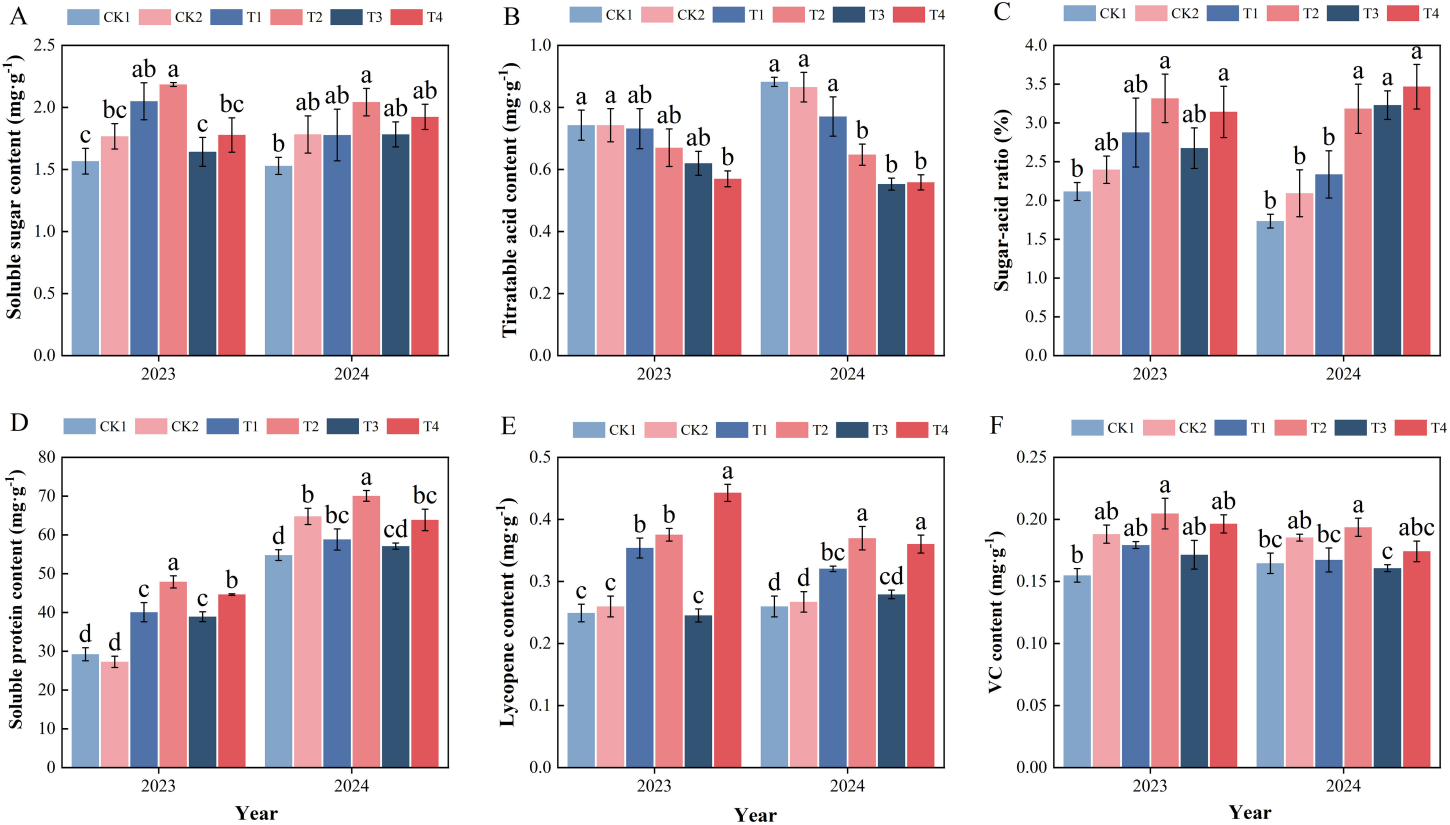
The effect of nitrogen application and companion cropping on tomato quality Over Two Years. **(A)** Soluble sugar content; **(B)** Titratable acid content; **(C)** Sugar-acid ratio; **(D)** Soluble protein content; **(E)** Lycopene content; **(F)** VC content. (CK1: No nitrogen application in tomato monoculture; CK2: No nitrogen application and companion cropping with potato onions; T1: Nitrogen reduction by 30% in tomato monoculture; T2: Nitrogen reduction by 30% and companion cropping with potato onions; T3: Conventional nitrogen application in tomato monoculture; T4: Conventional nitrogen application and companion cropping with potato onions. Different letters indicate significant differences, *P* < 0.05, same applies below).

The results in two years showed that the soluble sugar content of T2 tomatoes was significantly higher than that of CK1 (**Figure 2A**, *p* < 0.05). The titratable acid content of tomatoes in T4, T3, and T2 was lower than that in T1, CK1, and CK2, and reached a significant level in 2024 (**Figure 2B**, *p* < 0.05). Consistent with the trend observed for soluble sugars, the sugar-to-acid ratio of fruits from T2 and T4 was significantly higher than that of CK1 in both years (**Figure 2C**, *p* < 0.05). T2 had the highest soluble protein content, significantly higher than other treatments (**Figure 2D**, *p* < 0.05). The lycopene and VC content of T2 was found to be significantly higher than that of the control group, and the companion planting exhibited higher levels than monoculture cultivated at the same nitrogen level (**Figures 2E, F**, *p* < 0.05).

### 3.3 Effect of nitrogen reduction and potato onions companion cropping on enzyme activity of rhizosphere soil

**Figure 3:**
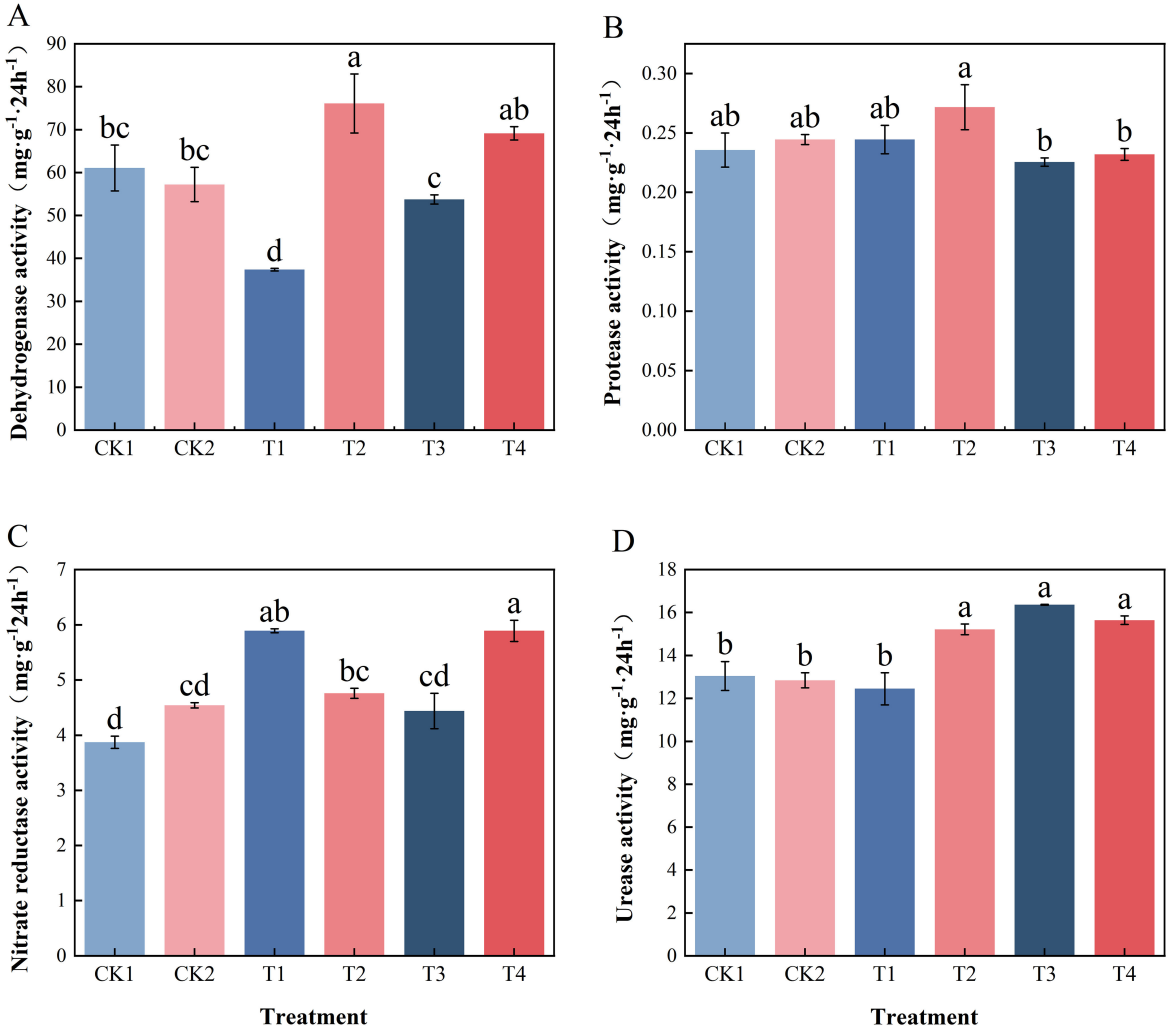
Effect of nitrogen application and companion cropping on enzymes activity related to nitrogen transformation in tomato rhizosphere soil. **(A)** Dehydrogenase activity; **(B)** Protease activity; **(C)** Nitrate reductase activity; **(D)** Urease activities. Different letters indicate the statistically significant differences among treatments (*P*<0.05).

Different nitrogen application rates and cultivation patterns significantly affected soil enzyme activity. The soil dehydrogenase activity of T2 was the highest, higher than that of T4, and significantly higher than that of CK1, CK2, T1 and T3 (**Figure 3A**, *p* < 0.05). Furthermore, under 30% nitrogen reduction and conventional nitrogen application levels, the companion cropping was significantly higher than the corresponding single cropping. The protease activity of T2 was found to be significantly higher than that of T3 and T4 (**Figure 3B**, *p* < 0.05), with no significant differences observed among the other treatments. Nitrate reductase activity exhibited its maximal level at T4, which was significantly higher than at T2, T3, and the control (**Figure 3C**, *p* < 0.05). Nitrogen application increased urease activity, with T3, T4, and T2 significantly higher than T1 and the control. Furthermore, at a 30% reduction in nitrogen, companion cropping was significantly higher than monoculture (**Figure 3D**, *p* < 0.05).

**Figure 4:**
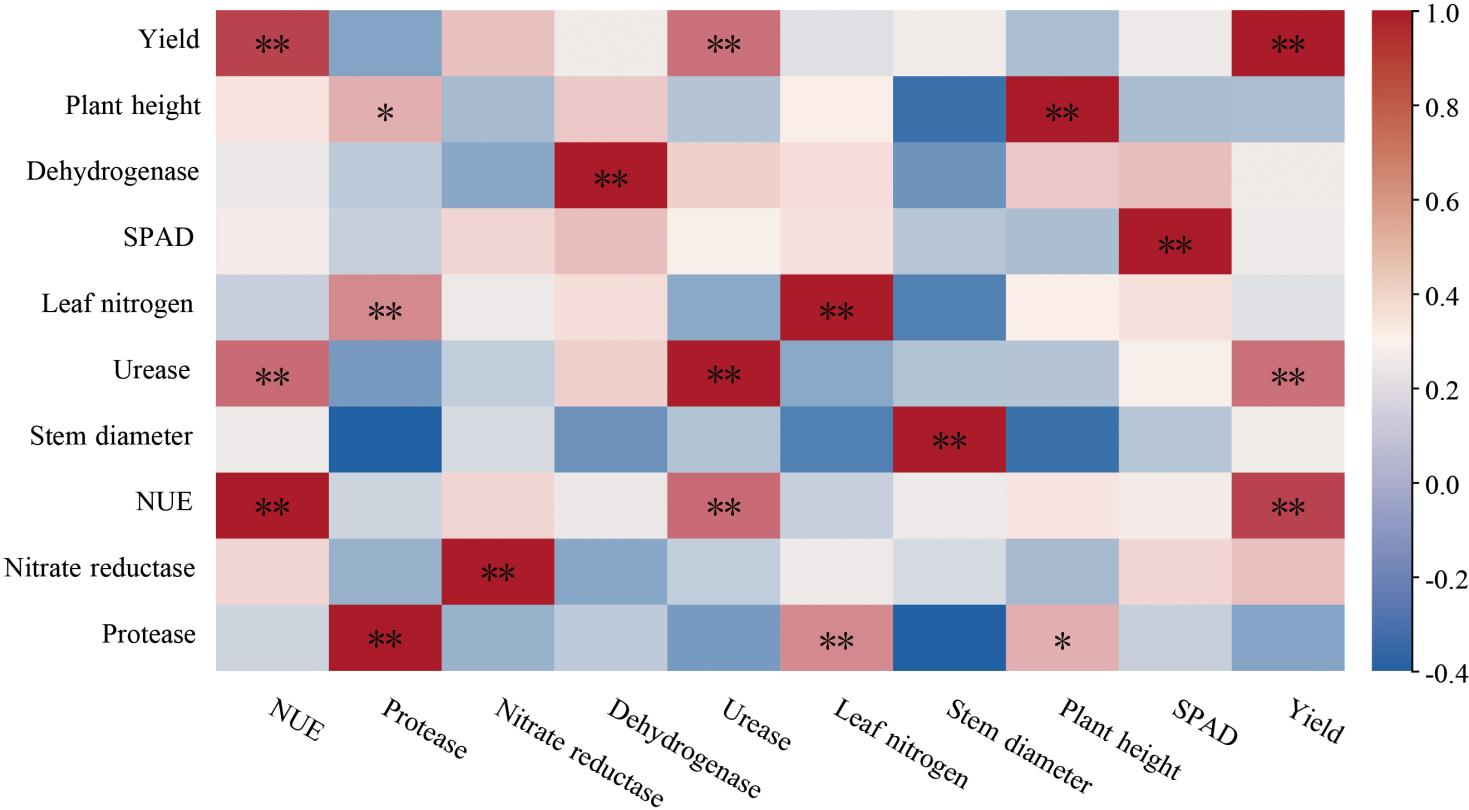
Correlation analysis of soil enzymes between tomato growth. (Pearson was used in correlation analysis. R> 0.5; *P < 0.05 significant correlation; **P < 0.01 highly significant correlation).

Correlation analysis was based on all treatments and nitrogen application levels, showing that soil urease activity was highly positively correlated with tomato NUE and yield (**Figure 4**, *p* < 0.01). Protease activity exhibited a highly significant positive correlation with leaf nitrogen content (*P* < 0.01) and a significant positive correlation with tomato plant height (*P* < 0.05). This finding suggests a close correlation between the activity of these two enzymes and tomato growth under nitrogen application and co-application patterns.

### 3.4 Effects of different nitrogen application levels and potato onions companion cropping on soil bacterial communities

#### 3.4.1 rhizosphere soil bacterial diversity and composition

**Figure 5:**
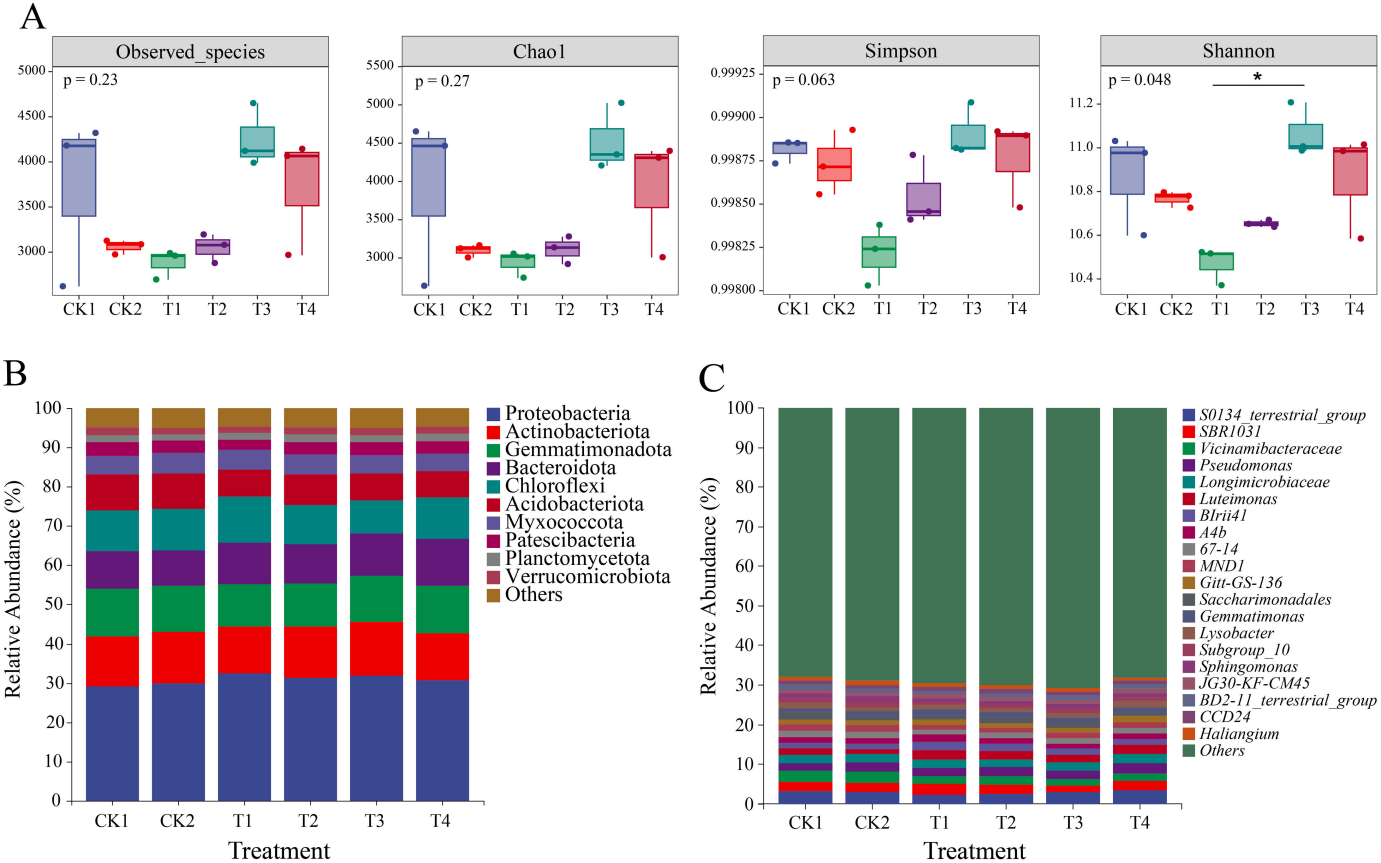
Effects of different nitrogen application levels and cultivation patterns on bacterial diversity and composition in tomato rhizosphere soil. **(A)** Bacterial Diversity Index; **(B)** Relative abundance of rhizosphere soil bacteria at phylum level; **(C)** Relative abundance of rhizosphere soil bacteria at genus level. (*P < 0.05 indicates significant difference between groups).

Nitrogen application rate and companion cropping altered soil bacterial community diversity(**Figure 5A**). T3 significantly increased the Chao 1 index, Shannon index, and Simpson index of soil bacterial communities compared with CK1 and T1. Compared with T1, the Shannon index was significantly increased (*p* < 0.05). A total of 48 bacterial phyla were identified, with the top 10 dominant soil bacterial phyla accounting for 95% of the total abundance (**Figure 5B**). Compared with CK1, T4 significantly increased the relative abundance of bacterial phyla such as Proteobacteria and Bacteroidota, and significantly decreased the relative abundance of bacterial phyla such as Acidobacteriota, Planctomycetota, and Verrucomicrobiota (*p*<0.05). With increasing nitrogen application, the abundance of Bacteroidota showed a gradual upward trend. Further comparison of the top 20 genera ranked by relative abundance (**Figure 5C**), as nitrogen application increased, the relative abundance of *Vicinamibacteraceae* decreased, while the relative abundance of *Pseudomonas, Longimicrbiaceae* and *Luteimonas* increased. Furthermore, under different nitrogen application levels, the relative abundance of *Pseudomonas* in the rhizosphere soil of the companion cropping was higher than that in the corresponding monoculture. The relative abundance of *MND1* decreased with increasing nitrogen application. Compared with CK1, the relative abundance of *Luteimonas* and *Gemmatimonas* significantly increased at a 30% nitrogen reduction level (*p* < 0.05).

#### 3.4.2 Rhizosphere soil bacterial community structure

**Figure 6:**
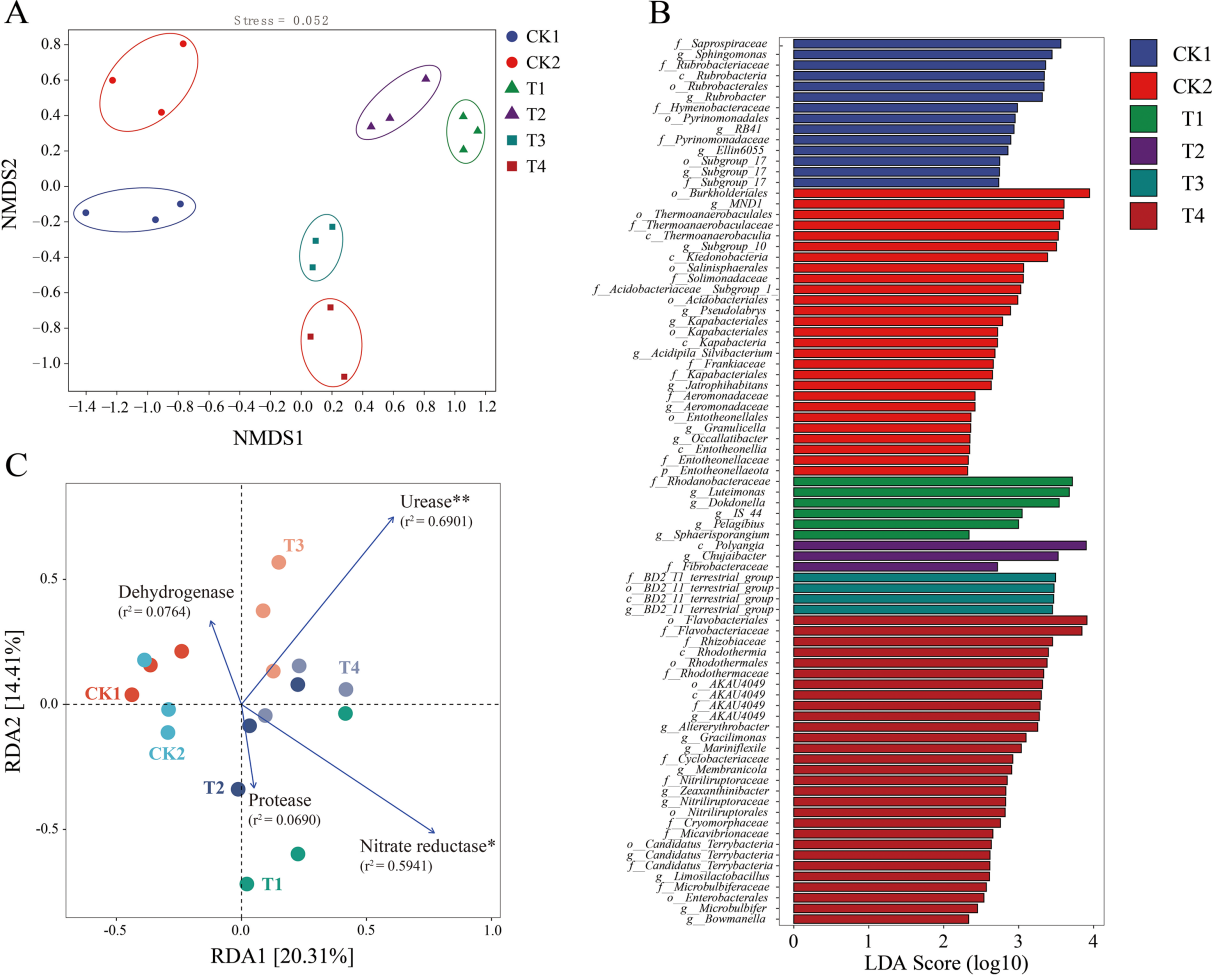
Effects of different nitrogen application levels and companion planting pattern on the community structure of rhizosphere soil bacteria and screening of differentially abundant Bacteria. **(A)** Non-metric multidimensional scale (NMDS) analysis of rhizosphere soil bacterial community structure in tomato based on Bray-curtis distance; **(B)** Bacterial LefSe analysis (LDA Score >2); **(C)** RDA analysis of rhizosphere soil bacterial community composition and Soil Enzymes (r^2^ >0.05). (*P < 0.05 indicates significant differences between groups; **P < 0.01 indicates highly significant differences between treatment groups).

NMDS analysis results showed significant differences in bacterial community structure between different treated soils (**Figure 6A**, ANOSIM, R1 = 0.851, *p* = 0.001; adonis, R2 = 0.444, *P* = 0.001). Lefse analysis clearly showed significant differences in the genus between different treatments (**Figure 6B**). A significant increase was observed in the abundance of *MND1*, *Subgroup_10*, *Burkholderiale*s, and *Salinisphaerales* in the CK2 samples when compared to the CK1 samples (*p* < 0.05). Different nitrogen application rates can enrich different microbial communities, with T4 showing the highest number of species, totaling 33, among which *Flavobacteriales* had the highest LDA value. The number of species differences in T2 was the lowest, with only 3 species, and the abundance of *Fibrobacteraceae*, *Polyangia*, and *Chujaibacter* was significantly higher than that of other treatments (*p*<0.05). RDA analysis was used to examine the relationship between changes in soil bacterial community structure and soil enzymes under different treatments (**Figure 6C**). Under different nitrogen application rates and cultivation modes, soil enzymes are closely related to bacterial communities. Furthermore, urease (r^2^ = 0.6901, *p* = 0.001) and nitrate reductase (r^2^ = 0.5941, *p* = 0.002) were the main factors influencing bacterial community structure.

### 3.5 Effect of nitrogen reduction and potato onions companion cropping on root exudates

#### 3.5.1 Root exudates composition

**Figure 7:**
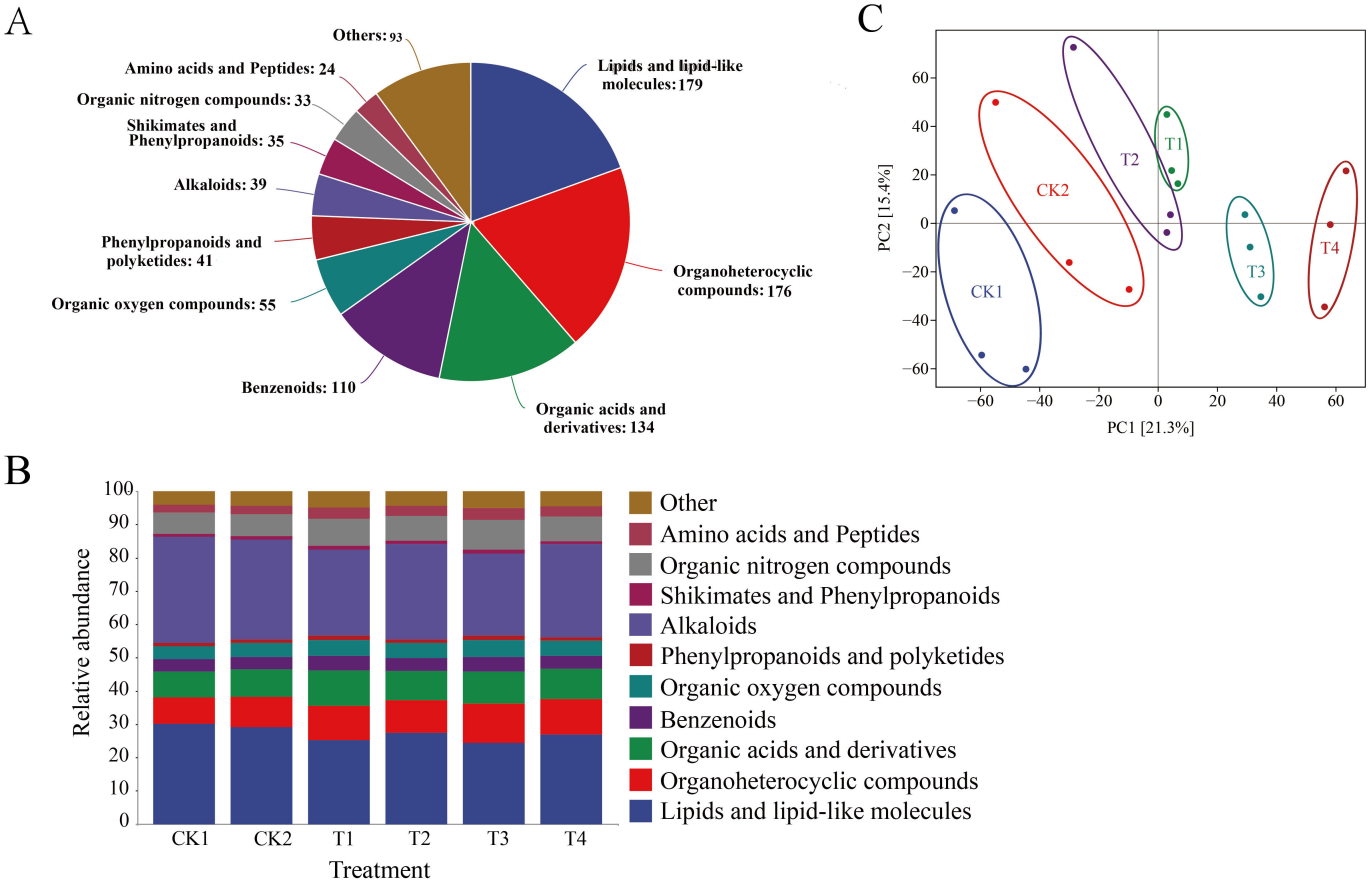
Effects of different nitrogen application levels and companion planting patterns on the composition of tomato root exudates. **(A)** Identification of root exudates; **(B)** Composition of root exudates under different treatments; **(C)** PCA of root exudates under different treatments.

A total of 919 metabolites were identified in tomato root secretions, including 179 Lipids and lipid-like molecules, 176 Organoheterocyclic compounds, 134 Organic acids and derivatives, 110 Benzenoids, 55 organic oxygen compounds, 41 Phenylpropanoids and polyketides, 39 Alkaloids, 35 Shikimates and Phenylpropanoids, 33 Organic nitrogen compounds, 24 Amino acids and Peptides, etc., which accounted for 89.9% of all root secretions9 (**Figure 7A**). Metabolite composition differs under different treatments. Compared with CK1, nitrogen treatment significantly reduced the relative abundance of lipids and lipid-like molecules and alkaloids. However, the relative abundance of organic nitrogen compounds gradually increased. Under the same nitrogen application rate, the relative abundance of lipids and lipid-like molecules and alkaloids in the root exudates of companion-planted tomatoes was significantly higher than that in monocultures (**Figure 7B**, *p* < 0.05). PCA analysis shows that PC1 and PC2 contribute 21.3% and 15.4%, respectively. This indicates that different treatments can alter the composition of tomato soil rhizosphere metabolites, and that nitrogen application rate has a greater impact on tomato root exudates than cultivation patterns (**Figure 7C**).

#### 3.5.2 Screening for differential root exudates

**Figure 8:**
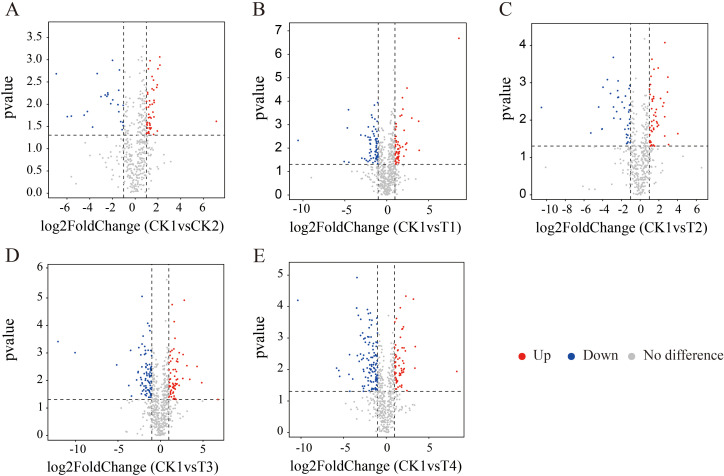
Screening of significantly different metabolites based on OPLS-DA model (Q2 > 0.5, VIP > 1 and *P* < 0.05). **(A)** Number of differentially metabolized compounds between CK1 and CK2, with blue indicating significant downregulation and red indicating significant upregulation; **(B)** Number of differential metabolites between CK1 and T1; **(C)** Number of differential metabolites between CK1 and T2; **(D)** Number of differential metabolites between CK1 and T3; **(E)** Number of differential metabolites between CK1 and T4.

Differential metabolite screening revealed 223 metabolites between CK2 and CK1 (**Figure 8A**), including 163 upregulated and 60 downregulated metabolites. Organoheterocyclic compounds accounted for the highest proportion of the upregulated metabolites (18.38%), while lipids and lipid-like molecules accounted for the highest proportion of the downregulated metabolites (30%). In monoculture, compared with CK1, T1 and T3 screened 327 and 365 differentially expressed metabolites, among which 192 and 201 were significantly upregulated, and 135 and 164 were significantly downregulated. The metabolites that were found to be up-regulated were primarily organoheterocyclic compounds and organic acids and derivatives, while those that were found to be down-regulated were chiefly lipids and lipid-like molecules (**Figures 8B, D**). Compared with CK1, T2 and T4 had 203 and 401 differentially expressed metabolites, respectively, among which 149 and 181 were significantly upregulated, and 81 and 220 were significantly downregulated (**Figures 8C, E**). Among the up-regulated metabolites, organoheterocyclic compounds were predominant, while the down-regulated metabolites were mainly lipids and lipid-like molecules.

#### 3.5.3 Differential root exudates and enrichment analysis

**Figure 9:**
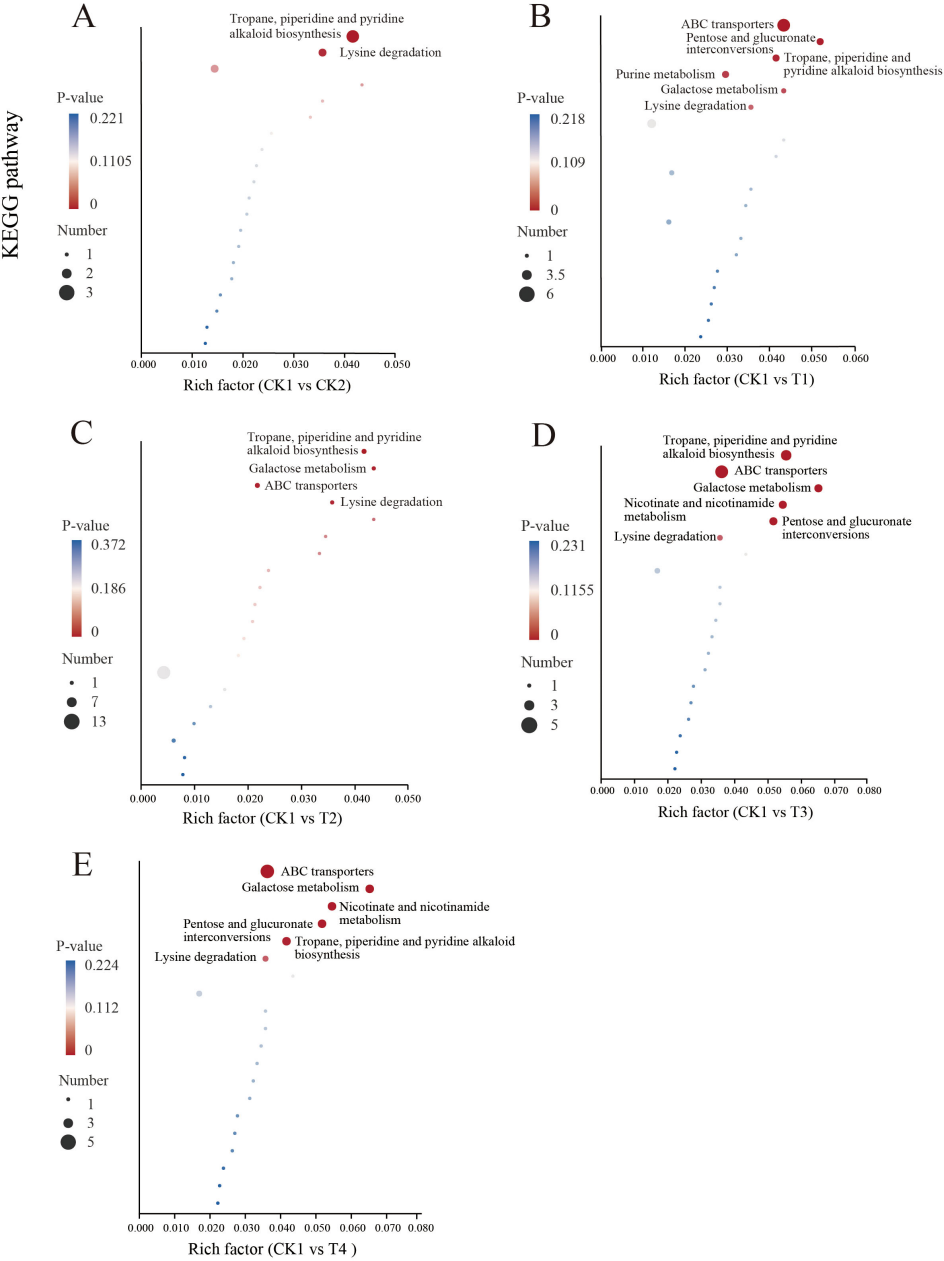
Metabolic pathway enrichment analysis of soil differential root exudates. **(A)** Differential metabolic and biosynthetic pathways enriched in CK1 versus CK2 comparisons (*P* < O.05); **(B)** Differential metabolic and biosynthetic pathways enriched in CK1 versus T1 comparisons (*P* < O.05); **(C)** Differential metabolic and biosynthetic pathways enriched in CK1 versus T2 comparisons (*P* < O.05); **(D)** Differential metabolic and biosynthetic pathways enriched in CK1 versus T3 comparisons (*P* < O.05); **(E)** Differential metabolic and biosynthetic pathways enriched in CK1 versus T4 comparisons (*P* < O.05).

**Figure 10:**
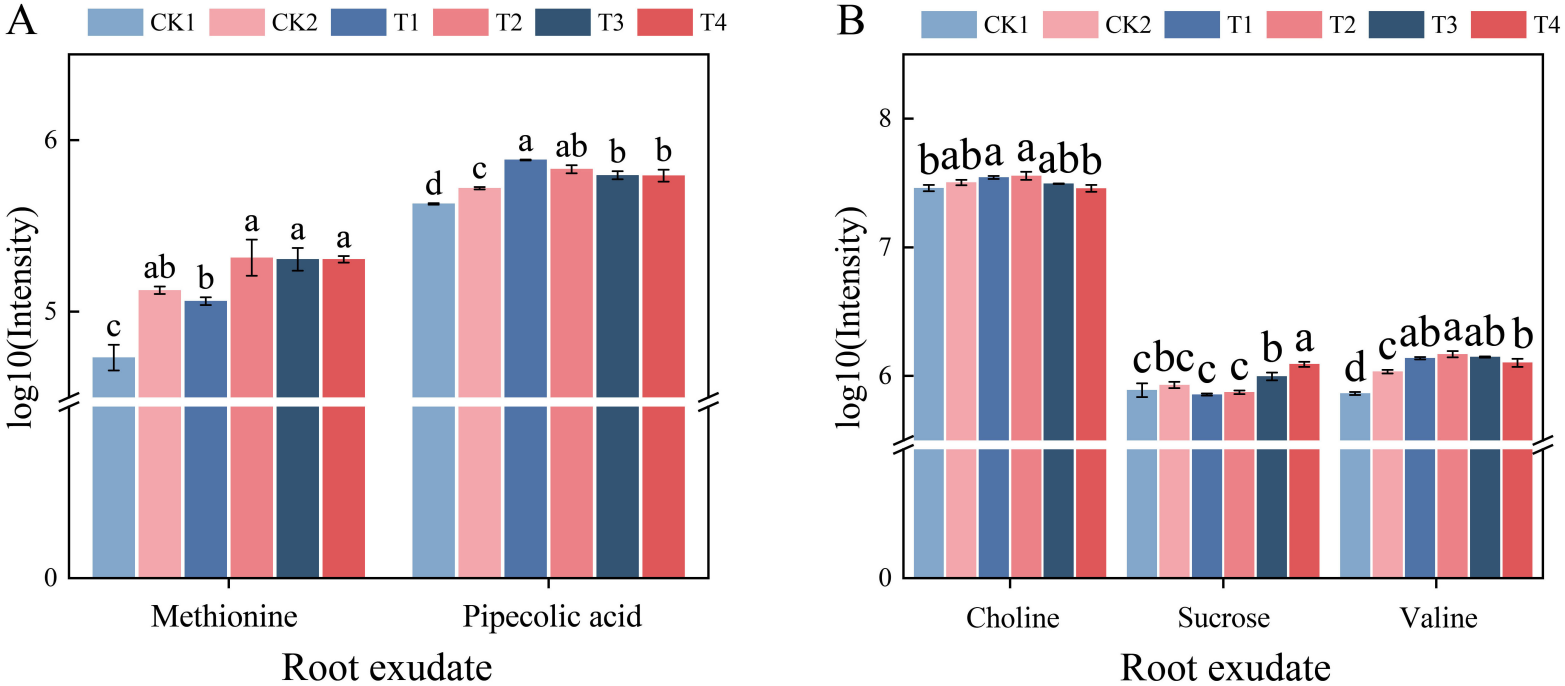
Effects of different nitrogen application rates and companion planting on the abundance of differential root exudates in tomato. **(A)** Differentially enriched metabolites in the lysine degradation pathway; **(B)** Differentially enriched metabolites in the ABC transporter pathway. Different letters indicate the statistically significant differences among treatments (*P*<0.05).

Through enrichment analysis of differential metabolite pathways, the specific changes in root-microbiome metabolic pathways were elucidated. In each treatment, the pathways of Tropane, piperidine, and pyridine alkaloids biosynthetic, ABC transporters, and Lysine degradation pathways were significantly enriched compared with the CK1 (**Figure 9**). In the Tropane, piperidine, and pyridine alkaloids biosynthesis, as well as Lysine degradation pathway, both monoculture and intercropping with different nitrogen application levels increased the abundance of pipecolic acid compared to the CK1. And the abundance of pipecolic acid was highest at T1, significantly higher than at T3, T4, and CK1 (**Figure 10A**, *p* < 0.05). Moreover, the abundance of methionine in T2 was significantly higher than that in T1 and CK1(*p* < 0.05). In the ABC transporters pathway, T1 and T2 were observed to significantly increase the abundance of choline and valine in comparison with CK1. Compared with CK1, CK2 significantly increased the abundance of valine (**Figure 10B**, *p* < 0.05). In comparison with CK1, T3 and T4 also significantly increased the abundance of sucrose in the ABC transporters pathway, with T4 being significantly higher than T3 (*p* < 0.05).

### 3.6 Correlation analysis of root secretion with soil bacterial community co-occurrence network and yield quality

**Figure 11:**
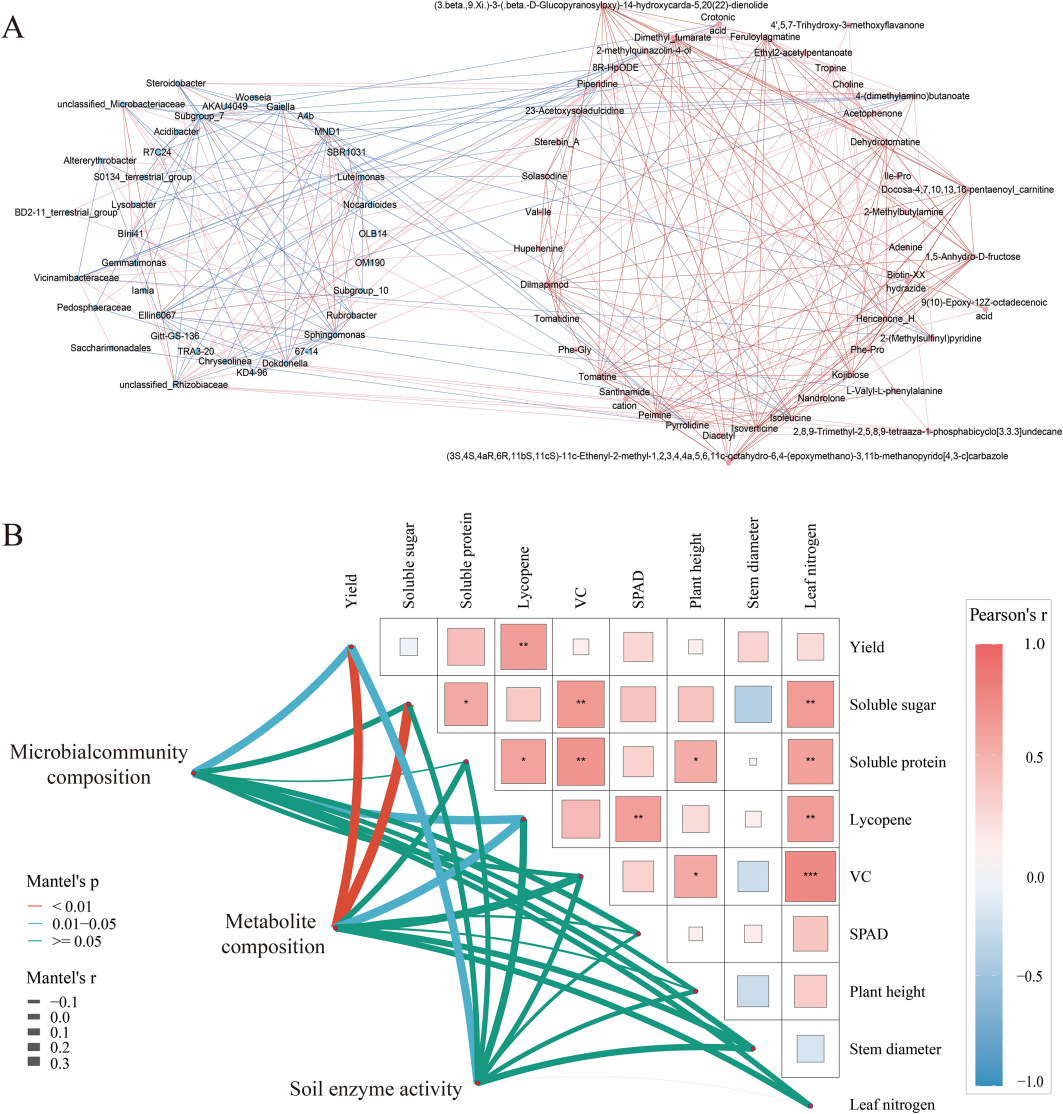
**(A)** Co-occurrence network analysis of rhizosphere soil differential metabolites and bacterial genera in tomato under different treatments (Red nodes represent different metabolites and blue nodes represent different bacterial taxa. Red lines indicate positive correlations and blue lines indicate negative correlations); **(B)** Mentel’s test and Pearson’s analysis of soil bacterial community composition, metabolite composition, and soil enzyme activities in relation to tomato growth and quality (Red lines indicate highly significant correlation, *p<*0.01; blue lines indicate significant correlation, *p* < 0.05). (* *P* < 0.05 indicates significant correlationin between dexes ; ** *P* < 0.01 indicates highly significant correlation between dexes; *** *P* < 0.001 indicates very highly significant correlation between dexes).

The co-occurrence network was constructed to elucidate the relationship between different treatments of tomato rhizosphere soil bacterial communities and root exudates. *MND1* was significantly positively correlated with bacterial genera such as *Sphingomonas, Rubrobacter, Ellin6067, and Subgroup_10*, in addition, it showed a significant negative correlation with metabolites such as pyrrolidine, piperidine, and isoleucine (**Figure 11A**, p < 0.05). In addition, piperidine showed significant positive correlations with bacterial genera such as *Steroidobacter* and *Chryseolinea*, as well as metabolites including pyrrolidine and crotonic acid (*p* < 0.05). The correlation analysis of tomato growth and quality demonstrated that tomato yield exhibited a positive correlation with stem thickness, leaf SPAD value, leaf nitrogen content, and soluble sugar content in fruits, and a significant positive correlation with lycopene content (**Figure 11B**, p < 0.05). Furthermore, the soluble sugar, soluble protein, lycopene, and vitamin C content of tomatoes showed a highly significant positive correlation with leaf nitrogen content (*p* < 0.01). Mantel test and Pearson analysis demonstrated that soil bacterial community composition, root exudate composition, and soil enzyme activity are the primary factors influencing tomato yield and quality. Among these, soil enzyme activity and soil bacterial community structure were significantly correlated with tomato yield (*p* < 0.05), and the composition of rhizosphere exudates was highly correlated with tomato yield and fruit soluble sugar content (*p* < 0.01). Moreover, bacterial communities and root exudates were significantly positively correlated with lycopene content in tomato fruits (*p* < 0.05).

## 4 Discussion

### 4.1 Nitrogen reduction and potato onions companion cropping for tomato growth and fruit quality improvement

Applying nitrogen is an effective means of promoting crops’ growth and increasing yields. Proper irrigation and nitrogen application can improve water and nitrogen resource utilization and reduce agricultural environmental pollution (Bai et al., 2023). Plants absorb nitrogen in three forms in nitrogen-fertilized soil: ammonium, nitrate and organic. These participate in nitrogen assimilation etc., thereby altering soil microbial diversity and promoting plant nitrogen uptake (Clouse and Wagner, 2021). Our research found that regardless of whether planted alone or in companion cropping, nitrogen application increased the SPAD value of leaves and the N content of plants, promoting plant growth and increasing tomato yield. In addition, under the same nitrogen application rate, the plant height, stem thickness, and SPAD value of leaves were higher in the companion planting than in the monoculture. This phenomenon may be attributed to the volatile organic compounds (VOCs) secreted by the potato onions, which have been observed to alter the rhizosphere environment of the tomato, significantly promoting tomato root growth and biomass accumulation (Shi et al., 2021). We also found that applying nitrogen fertilizers at appropriate rates (reducing nitrogen by 30%) can minimize nitrogen losses. Moreover, companion planting with potato onions promoted nitrogen absorption in tomatoes, thereby significantly improving nitrogen fertilizer utilization and increasing tomato yield. This is consistent with previous research findings that intercropping can promote plant growth (Zhou et al., 2011).

Soil enzyme activity can reflect the strength of soil biochemical activity and fertility. For example, urease plays a crucial role in nitrogen utilization in the soil nitrogen cycle (Zhang et al., 2004; Liu et al., 2008). In our study, nitrogen fertilizer reduction in monoculture system did not increase soil urease activity. This phenomenon may be attributed to the accelerated hydrolysis of urea under the high pH stress of saline-alkali soil, which inhibits urease activity in the alkaline environment, while the co-planted onion significantly increases urease activity. This may be related to changes in root exudates and soil environment caused by the co-planting (Chien et al., 2011). The implementation of a correlation analysis revealed a significant positive correlation between soil urease activity, tomato yield, and nitrogen utilization efficiency. Furthermore, protease activity exhibited a significant correlation with tomato plant height, suggesting a close relationship between the activity of soil urease and protease and tomato growth and nutrient absorption. Intercropping has been demonstrated to enhance the absorption of soil nutrients by crops, promote the accumulation of crop biomass, and improve the quality of the produce (Hoang et al., 2025). For example, in the intercropping system with garlic, the vitamin C content and soluble solids content of tomatoes were significantly increased (Liu et al., 2014). This is consistent with our research findings, which indicate that under companion planting conditions, the soluble sugar content, vitamin C content, and lycopene content of tomato fruits were significantly higher than those of tomatoes grown in monoculture. Compared with conventional nitrogen application with companion planting, reduced nitrogen application with companion planting significantly improved tomato nitrogen utilization efficiency and soluble protein content in tomato fruits. This validates our hypothesis that reducing nitrogen and using a companion planting model with onion can promote nitrogen absorption in tomatoes, thereby promoting growth and increasing yield, while also improving tomato quality.

### 4.2 The responses of rhizosphere microorganisms to nitrogen reduction and the companion cropping of potato onions

Soil bacteria have more nitrogen metabolism genes than fungi and archaea. They participate in all stages of nitrogen assimilation, nitrogen metabolism and nitrogen cycling. The nitrogen content in soil directly affects the community structure and composition of bacteria in soil (Li Y. et al., 2020). In our study, nitrogen application and tillering of onions altered the composition of soil bacterial communities. This finding aligns with previous research, which demonstrated that agricultural cropping patterns influence soil bacterial communities and their functions by altering the physical and chemical properties of the soil (Jiang et al., 2017). We found that at a 30% nitrogen reduction level, the diversity index of the bacterial community in the rhizosphere soil of tomatoes grown with potato onions was higher than that of tomatoes grown alone. The 30% reduction in nitrogen application reduced the diversity index compared to no nitrogen application, while bacterial communities were more diverse under conventional nitrogen application, which may be due to nitrogen-induced changes in nitrogen-sensitive microbial communities (Zhang L. et al., 2024). In intercropping system, soil microbial communities respond positively to fertilization levels (Ablimit et al., 2022). Following the application of nitrogen, a marked increase in the relative abundance of dominant bacterial phyla such as Bacteroidota and Proteobacteria was observed, whilst the relative abundance of the Acidobacteriota phylum and the genus *MND1* underwent a gradual decline. *Pseudomonas* is widely distributed in soil and plays a key role in promoting plant growth and enhancing resistance through its participation in denitrification, nitrogen fixation, nitrogen conversion, and dynamic effects on microbial communities. Meanwhile, increased nitrogen levels also affect the reproduction of *Pseudomonas* communities (Pham et al., 2017; Yang et al., 2024; Zhang et al., 2021). Certain Pseudomonas species, such as *Pseudomonas stutzeri*, is capable of biological nitrogen fixation and enhance plant nitrogen use efficiency (NUE) (Sanow et al., 2023; Li et al., 2025). In our study, nitrogen application increased the abundance of *Pseudomonas*, and at all nitrogen application levels, the abundance of *Pseudomonas* was higher in the companion planting than in the tomato monoculture. Intercropping has a positive effect on soil bacterial and fungal communities by promoting beneficial microorganisms and reducing certain potential plant pathogens (Yang X. et al., 2020). It is notable that the abundance of *MND1* decreased after nitrogen application, and this decrease was nitrogen concentration-dependent. This may be explained by the fact that higher soil salinity inhibits the nitrogen nitrification process in the soil, and *MND1*, which participates in the nitrogen cycle balance, is indirectly inhibited. On the other hand, excessive nitrogen levels can also directly inhibit the reproduction of the *MND1* bacterial community (Tang S. et al., 2023; Duan et al., 2025). Therefore, reducing nitrogen and improving the diversity of tomato soil microbial communities play a key role in improving nitrogen conversion in the soil.

### 4.3 The response of tomato root exudates to nitrogen reduction and companion cropping with potato onions

Plant root exudates are important mediators of plant-microbial interactions, and the diversity and composition of root exudates are closely related to plant diversity in intercropping systems. Intercropping has been demonstrated to have the capacity to modify the chemical diversity and composition of root exudates of the main crop (Zhu et al., 2023). For example, in a bean/wheat intercropping system, the composition of wheat root metabolites in the soil changes due to the nitrogen-fixing action of leguminous crops. The nitrogen-fixing factors synthesized by rhizobia promote the synthesis of flavonoids (Liu et al., 2017). Our study found that the composition of root exudates of tomatoes grown in companion cropping and monocropping systems differed significantly under different nitrogen application rates. In tomato monoculture system, as nitrogen application increases, the content of organic nitrogen compounds in the soil significantly increases. This may be because soil microorganisms drive soil nitrogen cycling and plant nitrogen uptake, playing a huge role in soil nitrogen assimilation (Canfield et al., 2010). At the same nitrogen application rate, companion planting increased the content of alkaloids in root exudates. This phenomenon may be attributed to the capacity of tomato roots to detect signals released by onion roots, such as dihydroquercetin, which induce alterations in their own root secretions. This alteration promotes the recruitment of beneficial microorganisms (such as *Pseudomonas*) in the tomato rhizosphere, thereby altering the secretion of their own alkaloids and improving tomato resistance (Wang et al., 2025; Wu et al., 2025). For example, within the tomato/potato intercropping system, the content of flavonoids in metabolites is observed to increase, thus promoting the colonization of beneficial microorganisms and consequently enhancing the disease resistance of tomatoes (Mathieu et al., 2024).

Under nitrogen stress, lysine-related metabolic pathways are significantly affected, indicating a dynamic interaction between lysine metabolism and nitrogen utilization (Song et al., 2023). In our study, Lysine degradation was significantly enriched after nitrogen application and companion planting, with pipecolic acid significantly enriched in this pathway and its abundance significantly increased compared to the control. Pipecolic acid has been demonstrated to reduce the carbon-to-nitrogen ratio of root exudates, which may consequently affect the nutrient utilization efficiency of plant roots (Preece et al., 2024). Nitrogen application and companion cropping also significantly increased the content of methionine in the rhizosphere. Research indicates that methionine can serve as a carbon and nitrogen source in soil, enhancing the activity of enzymes involved in nitrogen cycling, thereby increasing the activity of nitrogen-fixing bacteria and boosting the availability of essential nutrients in soil (Medina-de La Rosa et al., 2023; Wang et al., 2023). Furthermore, secretions such as methionine have been demonstrated to facilitate the dissolution of heavy metals within soil, thereby providing essential nutrients that exert a direct effect on the composition of the microbial community (Hao et al., 2022). Research has found that sucrose may stimulate the biosynthesis of nitrogen-containing compounds by activating nitrogen metabolism enzymes, including NR and NiR (Huang et al., 2022). Sucrose in pea nodules plays a crucial role in nitrogen fixation (Gordon et al., 1999). Under normal nitrogen application, the sucrose content in tomato root exudates significantly increases, potentially playing a crucial role in soil nitrogen transformation processes. It was shown that after exogenous selenium application, potatoes recruited beneficial bacteria such as *Bacillus* and *Pseudomonas* by secreting L-tyrosine, L-valine, and 4-oxoproline, thereby reducing nitrogen loss and improving nitrogen utilization (Liu et al., 2025). Therefore, companion planting altered the composition of metabolites by leveraging plant diversity. The enrichment of specific metabolites such as methionine, mucrose, and valine in tomato root exudates plays a crucial role in enhancing nitrogen utilization by recruiting beneficial soil microorganisms to colonize the rhizosphere. Further validation is required in the future.

### 4.4 Root exudates are closely associated with microbial communities, regulating nitrogen transformation and promoting tomato growth and nutrient absorption

Soil bacteria can influence crop root metabolism and soil fertility to varying degrees, while plant residues and root exudates in intercropping systems directly or indirectly affect soil bacterial community structure and play a key role in influencing total microbial biomass (Steinauer et al., 2016; Duchene et al., 2017). Research has confirmed that root exudates from intercropped tomatoes can activate various forms of phosphorus in the soil to enhance its availability, thereby promoting the aggregation of beneficial microbial communities such as *Pseudomonas* and *Bacillus* (Khashi U Rahman et al., 2021). Increasing evidence suggests that alterations in plants roots chemical composition can recruit beneficial microorganisms to plant rhizosphere, thereby promoting crop growth and nutrient uptake by influencing soil nitrogen cycling (Yuan et al., 2018; Wen et al., 2022). The collinearity network analysis in this study revealed a complex network of relationships between soil bacterial communities and root exudate formation under nitrogen reduction and cropping mode. Alkaloids such as Hupehenine and Solasodine were significantly positively correlated with the genus *Vicinamibacteraceae*, and the metabolite Tomatidine. And most root exudates are significantly positively correlated with each other. This indicates that there is a close relationship between soil root microorganisms and root exudates. Root exudates may activate microorganisms to improve soil fertility and soil environment. ABC transporters and the Lysine degradation pathway were significantly enriched under nitrogen-reduced fertilization, indicating an active response of the microbial interactions in tomato rhizosphere soil (Cox et al., 2019; Zhu et al., 2024). Our research results show that the composition of root exudates of companion planting tomatoes is significantly positively correlated with tomato yield and fruit soluble sugar content at different nitrogen application levels. Soil bacterial communities and soil enzymes are significantly correlated with tomato yield and lycopene content. This indicates that companion planting and fertilization can stimulate changes in root exudates, thereby affecting the structure of microbial communities and, consequently, tomato yield and quality (Zou et al., 2024). Based on these findings, our research revealed that the 30% reduction in nitrogen fertilizer application exhibits synergistic effects with companion planting, enhancing tomato nitrogen uptake by modulating interactions between soil root exudates and the microbial community. Nitrogen application induced the secretion of metabolites such as sucrose in tomatoes, while reduced nitrogen and companion planting promoted the accumulation of substances like Methionine and Valine in tomato exudates, thereby enhancing beneficial bacterial colonization. This cultivation model exerted a positive effect on both tomato yield and quality formation.

## 5 Conclusion

The results showed that, the 30% nitrogen reduction significantly promoted tomato growth in the companion planting pattern of tomato and potato onions. moreover, compared with conventional nitrogen application, tomato yield increased by 1.23% and 3.57%, and nitrogen use efficiency increased by 25.33% and 44.15%. The pattern of 30% reduction in nitrogen application with companion cropping increased the soluble sugar content and lycopene content of tomatoes significantly, and improved their quality. Nitrogen application enhanced urease activity, while nitrogen reduction and companion planting increased dehydrogenase and protease activity. The pattern of nitrogen reduction and companion planting enhanced tomato nitrogen uptake by regulating the accumulation of root exudates such as Sucrose, Methionine, and Valine in the soil. This process recruited beneficial microorganisms including *Pseudomonas*, *Luteimonas*, and *Gemmatimonas*, thereby promoting nitrogen utilization and ultimately increasing tomato yield. Under different cultivation systems, root exudates and soil microorganisms enhanced fruit quality by increasing soluble sugar and lycopene content in the fruit.

## Data Availability

The original contributions presented in the study are publicly available. This data can be found here: National Center for Biotechnology Information (NCBI) BioProject database under accession number PRJNA1355305.
